# Functional hemichannels formed by human connexin 26 expressed in bacteria

**DOI:** 10.1042/BSR20140089

**Published:** 2015-03-18

**Authors:** Mariana C. Fiori, Srinivasan Krishnan, D. Marien Cortes, Mauricio A. Retamal, Luis Reuss, Guillermo A. Altenberg, Luis G. Cuello

**Affiliations:** *Department of Cell Physiology and Molecular Biophysics, Texas Tech University Health Sciences Center, Lubbock, TX 79430-6551, U.S.A.; †Centro de Fisiología Celular e Integrativa, Facultad de Medicina, Clínica Alemana, Universidad del Desarrollo, Avenida Las Condes 12438, Lo Barnechea, Santiago, Chile; ‡Center for Membrane Protein Research, Texas Tech University Health Sciences Center, Lubbock, TX 79430-6551, U.S.A.

**Keywords:** adenosine 5′-triphosphate (ATP), calcium, connexon, deafness, gap-junction channel, method, AF350, Alexa Fluor 350, AF647, Alexa Fluor 647, Cx26, connexin 26, DDM, *n*-dodecyl-β-D-maltoside, GJC, gap-junction channel, HC, hemichannel or connexon, NLM, sodium liquid medium, PC, phosphatidylcholine, PE-R, phosphatidyl-ethanolamine labelled with lissamine rhodamine B at the headgroup, PS, phosphatydilserine, TEV, tobacco etch virus

## Abstract

Gap-junction channels (GJCs) communicate the cytoplasm of adjacent cells and are formed by head-to-head association of two hemichannels (HCs), one from each of the neighbouring cells. GJCs mediate electrical and chemical communication between cells, whereas undocked HCs participate in paracrine signalling because of their permeability to molecules such as ATP. Sustained opening of HCs under pathological conditions results in water and solute fluxes that cannot be compensated by membrane transport and therefore lead to cell damage. Mutations of Cx26 (connexin 26) are the most frequent cause of genetic deafness and it is therefore important to understand the structure–function relationship of wild-type and deafness-associated mutants. Currently available connexin HC expression systems severely limit the pace of structural studies and there is no simple high-throughput HC functional assay. The *Escherichia coli*-based expression system presented in the present study yields milligram amounts of purified Cx26 HCs suitable for functional and structural studies. We also show evidence of functional activity of recombinant Cx26 HCs in intact bacteria using a new growth complementation assay. The *E. coli*-based expression system has high potential for structural studies and high-throughput functional screening of HCs.

## Abstract 2

Human connexin 26 (Cx26) hemichannels (HCs) can be expressed in bacteria in a functional form. This new expression system yields milligram amounts of purified HCs and has high potential to advance studies of connexin HCs.

## INTRODUCTION

Gap-junction channels (GJCs) are formed by head-to-head association of hemichannels (HCs, connexin hexamers, connexons), one from each of the neighbouring cells [1–3]. GJCs are aqueous channels that mediate electrical and chemical coupling between cells due to their insulation from the extracellular fluid and their permeability to hydrophilic molecules of up to 400–800 Da, depending on the isoform [2,4,5]. Undocked HCs that are ’free’ at the plasma membrane (not forming GJCs, referred to as HC hereafter) communicate two compartments of very different composition (intracellular and extracellular fluids). Even though HCs are mostly closed, they still play roles in physiologic processes by mediating the transmembrane fluxes of hydrophilic molecules such as ATP, NAD^+^, glutamate, glutathione and prostaglandin E_2_ [6–9]. However, sustained opening of HCs under pathological conditions (e.g., during ischaemia/hypoxia) results in water and solute fluxes that cannot be compensated by the cells (metabolite loss, Ca^2+^ influx, equilibration of ionic gradients, colloid-osmotic cell swelling) and lead to cell damage [10–13].

Approximately 1/1000 infants have profound hearing impairment and a large fraction of these cases can be ascribed to Cx26 (connexin 26) mutations [5,14,15]. Significant progress has been made in recent years, but the information available is insufficient to understand the mechanisms of the functional effects of most of the mutations associated with deafness [5,14].

Experiments ’*in vivo*’ are fundamental to understand biological processes, but ‘*in vitro*’ studies using isolated systems under well-controlled conditions are also an essential component for a complete understanding of normal function and the molecular mechanisms of diseases. Most frequently recombinant connexins are expressed in mammalian cell lines, insect cells and frog oocytes. The insect-cell–baculovirus expression system is the only available system that can yield the amounts of purified connexins necessary for detailed biochemical and biophysical studies [16–20]. In the present paper, we present a Cx26 *E. coli*-based expression–purification system that also yields milligram amounts of functional human Cx26 HCs.

## MATERIALS AND METHODS

### Protein expression and purification

**Figure 1:**
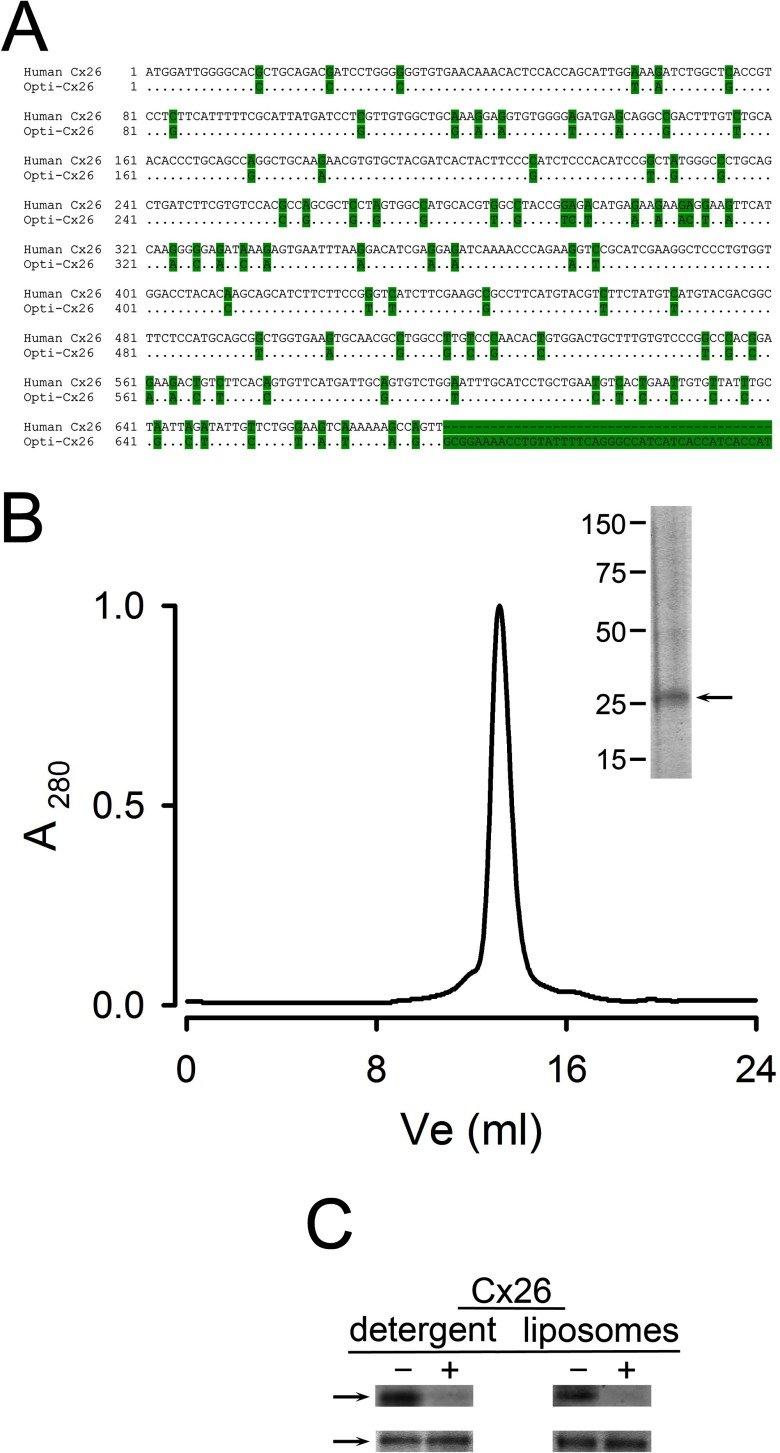
Recombinant human Cx26 purified from *E. coli* (**A**) DNA sequence of the human Cx26 used for *E. coli* expression. Comparison of the human Cx26 gene and the optimized (Opti-Cx26) sequence used in the present work. The highlighted bases indicate the mutations introduced in the gene. The final unmatched sequence codes for the TEV protease sequence and the poly-histidine–tag. (**B**) Size-exclusion chromatogram of purified Cx26. Approximately 250 μg of purified Cx26 solubilized in DDM was injected into the column. Inset: Coomassie Blue-stained gel of the peak fraction (∼10 μg of protein). The positions of molecular-weight markers are indicated on the left. The arrow points to Cx26 whose identity was confirmed by Western blots with anti-Cx26 and anti-histidine antibodies. (**C**) Orientation of Cx26 HCs reconstituted in liposomes. The C-terminal histidine–tag accessibility was assessed by comparing samples in the absence of TEV protease (−) and after treatment with the enzyme [+,1:10 (w/w) ratio, overnight at room temperature]. HCs (typically 20 μg of protein) were studied solubilized in detergent or reconstituted in liposomes. Top: Immunoblots using an anti-histidine–tag antibody (anti-Penta-His, Qiagen). Bottom: Coomassie Blue-stained bands showing similar levels of protein. The data are representative of six similar experiments.

We expressed and purified wild-type human Cx26 fused to a poly-histidine–tag [His_6_] at the C-terminal end, preceded by a TEV (tobacco etch virus) protease cleavage sequence [21]. XL10-Gold cells (Agilent Technologies) were transformed with human Cx26 DNA subcloned into the *Nco*I/*Hin*dIII sites of the bacterial protein expression vector pQE60 (Qiagen). Expression from the human Cx26 DNA sequence was very low and difficult to detect, but expression from the *E. coli*-optimized Cx26 sequence in Figure 1(A) was higher (pQE–Cx26 plasmid). The cells were grown at 37°C in modified M9 minimal medium (180 mM Na_2_HPO_4_, 75 mM KH_2_PO_4_, 30 mM NaCl and 65 mM NH_4_Cl) supplemented with 10 mM MgSO_4_, 1% glucose and 0.4 mg/ml ampicillin. The overnight cultures were diluted 25-fold, grown at 37°C to an *A*_600_ ∼2 and induced with 0.5 mM IPTG (0.5 mM) for 2 h. Harvesting of the cells and all subsequent procedures were performed at 4°C unless specified otherwise. The cell pellets were resuspended in buffer A (300 mM NaCl and 50 mM Tris/HCl, pH 8) with 0.5 mM 4-benzenesulfonyl fluoride hydrochloride (Pefabloc), 10 mM MgCl_2_ and 25 μg/ml DNAse I (Sigma–Aldrich) and lysed on a microfluidizer. Crude membranes were prepared by centrifugation at 100000 ***g*** for 1 h. The efficiency of the solubilization of Cx26 from membranes was determined by Western blotting, comparing the amounts of Cx26 in the supernatants (solubilized material) and pellets after centrifugation at 100000 ***g*** for 30 min. Membranes were solubilized for 4 h at 4°C with 1% Anzergent 3–12 in 1 M NaCl, 50 mM Tris/HCl, 10% glycerol and 1 mM PMSF, pH 8, at a total protein concentration <2 mg/ml. Following ultracentrifugation at 100000 ***g*** for 30 min, the solubilized material in the supernatant was loaded onto a Talon Co^2+^ column (Talon Superflow, Clontech) pre-equilibrated with 1 M NaCl, 10% glycerol, 50 mM Tris/NaOH, pH 8, for immobilized metal-affinity chromatography (IMAC). The protein-bound resin was washed with 10 column volumes of 1 M NaCl, 10% glycerol, 0.05% *n*-dodecyl-β-D-maltoside (DDM), 50 mM Tris/NaOH, pH 8, followed by washing with 150 mM NaCl, 10% glycerol, 5 mM imidazole, 0.05% DDM and 50 mM Tris/NaOH, pH 8. Elution proceeded with a buffer of the same composition, except that imidazole was increased to 300 mM. Fractions containing Cx26 were pooled and, in most cases, the histidine–tag was removed by incubation with TEV protease (1:10 w/w) for 12 h, at 4°C. After removal of the tag, purified Cx26 was isolated by gel-filtration chromatography on a Superdex 200HR column (GE Healthcare) run on an APLC system (LabAlliance, State College). Basically, IMAC-purified Cx26 in 0.05% DDM, 150 mM NaCl, 10% glycerol and 10 mM HEPES/NaOH, pH 7.5, was injected into the column equilibrated with the same buffer and run at a flow rate of 0.5 ml/min. The oligomerization of solubilized Cx26 was determined as previously described [22], by gel filtration and dynamic light scattering measured at 90° using a BI-200SM (Brookhaven Instruments). Protein concentrations were determined from the *A*_280_ nm.

### Reconstitution of Cx26 HCs

Reconstitution was performed in a mixture of phosphatidylcholine (PC) and phosphatidylserine (PS) at a 2:1 ratio (w/w), essentially as described [20,22]. Briefly, lipids and solubilized Cx26 were mixed, generally at a protein–lipid ratio of 1:50 (w/w), and the mixture was run through a gel-filtration column (Zeba columns, Thermo Fisher Scientific) pre-equilibrated with a solution containing 100 mM KCl, 0.1 mM EGTA and 25 mM HEPES, pH 7.6. In some experiments, this procedure was followed by extrusion (Mini-Extruder, Avanti Polar Lipids). For the sucrose-transport experiments, the liposomes were prepared as described [20,22] and contained traces of phosphatidylethanolamine headgroup labelled with lissamine rhodamine B (PE-R, PC–PS–PE-R ratio of 2:1:0.03, w/w) and the buffer composition was: 10 mM KCl, 0.1 mM EDTA, 0.1 mM EGTA, 459 mM urea and 10 mM HEPES, pH 7.6.

### Transport assays

Permeabilities to sucrose and the fluorescent probes Alexa Fluor 350 and 647 (AF350 and AF647 respectively) were determined by the transport-specific assay [20,22,23]. Liposomes and proteoliposomes were loaded with 500 μM AF350 and 125 μM AF647 and after removal of most extraliposomal dyes by gel filtration chromatography (Superdex 200 5/150 GL column, GE Healthcare) the samples were centrifuged on an iso-osmolar (sucrose/urea) gradient. The position of the liposomes was determined by the *A*_280_ nm or the fluorescence from PE-R. We have demonstrated that the migration of the liposomes down the sucrose gradient is the result of the presence of HCs [20]. Retention of dye trapped in liposomes was detected at excitation and emission wavelengths of 345 and 445 nm for AF350 and 650 and 670 nm for AF647. Details on the transport assays for ATP, Ca^2+^ and H^+^ equivalents have been published [22]. Basically, liposomes were loaded with the low-affinity Ca^2+^-sensitive probe Fluo-5N (pentapotassium salt, 25 μM, Life Technologies) for the Ca^2+^ influx assays, followed by removal of the extraliposomal probe by gel filtration, as described above. Since Fluo-5N is too large to permeate through HCs (958 Da) it remains inside the liposomes independently of the presence or absence of Cx26 HCs. For the determination of the Ca^2+^ influx, free-[Ca^2+^] was increased from <10 nM to 500 μM by rapid-mixing on a SX20 stop-flow device with a 20-μl chamber and a dead time <0.5 ms (Applied Photophysics). We have shown that the increase in Fluo-5N emission results from the Ca^2+^ influx through HCs and analysis of the rate of fluorescence increase can be used to estimate Ca^2+^ permeability [22]. Transport of H^+^ equivalents (H^+^/OH^−^ and protonated/unprotonated HEPES) was determined from the rate of intraliposomal pH changes in liposomes containing traces of a phospholipid labelled with fluorescein at the headgroup. The fluorescein-labelled phospholipid was *N*-(fluorescein-5-thiocarbamoyl)-1,2-dihexadecanoyl-*sn*-glycero-3-phosphoethanolamine (DHPE-Fl, headgroup–labelled, Life Technologies), used at a PC–PS–DHPE-Fl ratio of 2:1:0.005 (w/w). Starting from an identical solution inside and outside the liposomes (100 mM KCl, 0.1 mM EGTA, 25 mM HEPES/KOH, pH 7.6), extraliposomal pH was reduced to 6.4 by mixing with HCl in the stop-flow chamber. We have shown that the decrease in the fluorescence from the fluorescein-labelled phospholipids is linear with lowering pH from 7.6 to 6.4 and the influx of H^+^ equivalents can be calculated from the initial rate of pH change and the buffer concentration and pKa. For the Ca^2+^ and pH studies excitation was at 470 nm and emission was collected through a 500-nm long-pass filter, with all experiments were performed at 20°C.

### Analysis of Cx26 HC function in intact bacteria

For these studies, we developed a simple growth complementation assay using LB2003 cells. LB2003 cells deficient in K^+^ uptake mechanisms (knockout of the major uptake mechanisms Kdp, Kup and Trk) were generously provided by Dr E.P. Bakker from Osnabrük University in Germany [24]. These cells cannot grow in low-[K^+^] medium, but growth can be restored by expression of K^+^ channels or supplementation of the medium with K^+^ [25–28]. Since HCs are poorly-selective channels that permeate K^+^, among other ions [4], we determined whether their expression allowed LB2003 cells to grow in low-[K^+^] medium. Competent LB2003 cells transformed with the empty pQE60 plasmid or the plasmid containing the human Cx26 DNA were grown overnight in Luria–Bertani (LB) medium supplemented with 100 mM K^+^ and 400 μg/ml ampicillin. The cells were washed three times in low-[K^+^] medium [23 mM NaH_2_PO_4_, 46 mM Na_2_HPO_4_, 8 mM (NH_4_)_2_SO_4_, 0.4 mM MgSO_4_] and were then resuspended to an *A*_600_ of 0.2 in the same low-[K^+^] supplemented with 1 mM sodium citrate, 8 mM glucose and 1 mg/l thiamine B1 [sodium liquid medium (NLM)] supplemented with 400 μg/ml ampicillin and 0.5 mM IPTG. We then seeded 3 ml of samples in 24-well plates (931565-G-1X, Thompson Scientific Co.), incubated the plates with shaking at 250 rpm in an incubator and assessed cell growth from the *A*_600_ after 18 h (growth reached fairly stable levels between 16 and 26 h). Studies on the dependence of cell growth on medium [K^+^] showed 50% of maximal growth at ∼30 mM [K^+^] and based on these results we used the NLM supplemented with 4 mM K^+^ for the growth complementation assay. The initial *A*_600_ of 0.2 represented no growth and was subtracted from all values.

### Western blots

Cx26 expression was assessed from Western blots of cell lysates using antibodies against the histidine–tag (anti-Penta-His, Qiagen) or the Cx26 intracellular loop region (Life Technologies). Detection in Western blots was by imaging (Odyssey Infrared Imager, Li-Cor Biosciences) of goat anti-rabbit IRDye 800 (Li-Cor Biosciences) for the anti-Cx26 antibodies or goat anti-mouse Alexa Fluor 680 (Life Technologies) for the anti-histidine antibody.

### Statistics

Data shown are means ± S.E.M. Statistically significant differences were assessed by the Student's *t* test for unpaired data or one-way ANOVA, as appropriate.

## RESULTS AND DISCUSSION

There are very few studies describing the expression of connexins in *E. coli* [29,30]. In one study, human Cx43 fused to GST was purified, but transport function was not assessed [29]. Although not explored, it seems likely that the preparation consisted of purified inside-out membranes containing the Cx43 fusion protein because detergents and centrifugation procedures to separate membranes from soluble proteins were not used [29]. In another study, human Cx26 and rat Cx46 were expressed in *E. coli* [30]. In that study, a human Cx26 gene without optimization for expression in *E. coli* was used, the expression conditions were different and a strong anionic detergent (N-lauroylsarcosine) was employed, with the resulting recovery of connexins as monomers. In our study, we aimed at purifying functional Cx26 HCs, as we have previously done from Cx26 expressed in Sf9 cells [22]. Using Anzergent 3–12 we were able to solubilize <50% of the Cx26 expressed in membranes, but essentially all was present as HCs, similar to Cx26 purified from insect cells [22]. Cx26 expressed in *E. coli* (Figure 1A) was purified by metal affinity chromatography based on the C-terminal histidine–tag, followed by size-exclusion chromatography. Figure 1(B) shows a gel filtration chromatogram of the purified protein and the inset corresponds to a Coomassie Blue-stained gel of the peak fraction. Overloaded gels (standard denaturing and reducing SDS/PAGE) show several bands corresponding to monomer and oligomers. This is the result of the high-stability of purified Cx26 oligomers that has been observed before [22]. However, dynamic light scattering of the protein purified from *E. coli* in detergent solution showed a single peak corresponding to a hydrodynamic radius of 5.3±0.3 nm (*n*=4) and an apparent molecular weight of 230±10 kDa by size-exclusion chromatography. These values are indistinguishable from those of human Cx26 produced using the baculovirus–Sf9 cells expression system [22]. The apparent size of the protein–detergent complex is compatible with Cx26 HCs (hexamers) with 30%–45% (w/w) detergent, but not with GJCs (dodecamers) [22]. The yield of purified Cx26 HCs was 0.25–0.5 mg/l of cell culture.

**Figure 2:**
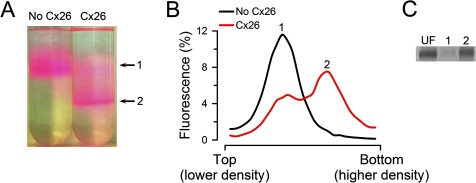
Permeability of purified and reconstituted Cx26 HCs to sucrose (**A**) Typical migration of liposomes and proteoliposomes containing Cx26 HCs on an iso-osmolar sucrose-density gradient. Picture of tubes after centrifugation showing liposomes without HCs (upper arrow, labelled 1) and proteoliposomes with functional HCs (lower arrow, labelled 2). The lipid–protein ratio for reconstitution was 1:50 (w/w). The colour of the liposomes is due to traces of PE-R. (**B**) Fluorescence from rhodamine B-labelled lipids measured in equal-volume fractions collected from liposomes (No Cx26) and proteoliposomes (Cx26) gradients in panel (**A**). Rhodamine B fluorescence was normalized to the total fluorescence. The 1 and 2 labels indicate the peak fluorescence corresponding to the bands labelled in panel (**A**). (**C**) Distribution of reconstituted Cx26 in sucrose-permeable and -impermeable liposomes. Cx26 was reconstituted and run on an iso-osmolar sucrose gradient [see Cx26 tube on panel (**A**)]. Equal volumes of fractions including the bands labelled 1 (liposomes impermeable to sucrose) and 2 (liposomes permeable to sucrose) were run on an SDS/PAGE and stained with Sypro Ruby. UF, equivalent amount of unfractionated liposomes. The gel is representative of four similar experiments. In all cases, the HCs studied were formed by Cx26 without the C-terminal poly-histidine–tag.

To assess the proportion of HCs in the cytoplasmic-side-out (inside-out) orientation, we took advantage of the TEV protease cleavage site, located immediately N-terminal to the poly-histidine–tag, to remove the tag by proteolysis. Figure 1(C) shows that essentially all the histidine–tag signal disappeared when Cx26 solubilized in 0.05% DDM (100±0%, *n*=4) or reconstituted (100±0%, *n*=6) was digested with TEV protease. Therefore, it appears that all HCs insert inside-out. This is similar to Cx43 [20,31], but different from the approximately random insertion that we found in a previous work on Cx26 [22]. Since the lipids are the same, the difference is probably the result of the method of reconstitution; dialysis in the previous work [22] and/or gel-filtration/extrusion here. The precise reason for the difference was not explored, but a single orientation is clearly an advantage for some studies.

**Figure 3:**
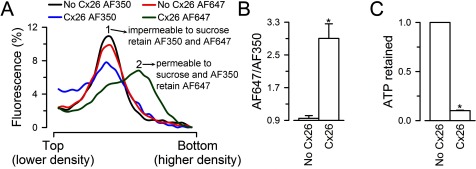
Permeability of purified and reconstituted Cx26 HCs to fluorescent dyes and ATP (**A**) Permeability to Alexa Fluor probes. The liposomes were loaded with AF350 or AF647. After removal of the free extraliposomal dyes by size-exclusion chromatography, the liposomes were subjected to an iso-osmolar sucrose-density gradient such as that in Figure 2(A) and the samples were analysed as in Figure 2(B), for AF350 and AF647 fluorescence. The fluorescence of each fraction was expressed as a percentage of the cumulative fluorescence of all fractions. AF350 and AF647 data are representative from four and seven similar experiments respectively. (**B**) Ratio of AF647–AF350 fluorescence in Cx26 proteoliposomes relative to the liposome value. Data are means ± S.E.M. (*n*=4). Values were calculated from experiments similar to those in panel (**A**). The AF647–AF350 ratio was obtained by dividing the AF647 fluorescence (probe retained in no Cx26 and Cx26 liposomes) by the AF350 fluorescence (probe lost from the Cx26 liposomes). *P<0.001 compared with the liposome values. (**C**) Permeability of purified Cx26 HCs to ATP. Liposomes and Cx26 proteoliposomes loaded with 1 mM ATP were subjected to gel filtration to separate free extraliposomal ATP from ATP retained inside the liposomes. ATP retained was measured by luminescence using a luciferin/luciferase assay, as the difference between total ATP (inside plus outside, measured after addition of Triton X-100 to 0.4%) and extraliposomal ATP (before Triton X-100). Data are expressed as means ± S.E.M. of the ATP retained/total ATP (*n*=4). *P<0.001 compared with the liposome values. In all cases, HCs were formed by Cx26 without the C-terminal poly-histidine–tag.

The Cx26 HCs expressed in bacteria were permeable to sucrose (Figures 2A and 2B) as well as to AF350, but not to AF647 (Figures 3A and 3B). For these studies, we used the transport-specific fractionation technique developed by Harris and Bevans [23], which allows for the separation of sucrose-permeable from sucrose-impermeable liposomes [20,22]. Upon centrifugation on an iso-osmolar sucrose gradient ([sucrose] increasing from top to bottom, compensated by a reversed urea gradient to maintain the osmolarity constant at all levels), the heavier sucrose-loaded liposomes that contain sucrose-permeable HCs, migrate as a narrow band to a higher-density (lower) position in the tube (Figure 2A, arrow labelled 2). There was no evidence for a significant population of non-functional HCs since essentially all Cx26 was localized to the higher-density band (band 2) of the sucrose gradient, with a very small fraction at the lower-density upper band (Figures 2B and 2C).

For the Alexa-dyes permeability assays, we loaded AF350 and AF647 into the liposomes, removed the extraliposomal fluorescent probes by size-exclusion chromatography and then performed a transport-specific fractionation as described above. Fluorescence was measured in equal-volume aliquots from the lower-density (top of tube) to the higher-density (bottom of tube) fractions to determine whether the probes remained inside the liposomes (impermeable) or leaked out through the HCs during the gel filtration and/or centrifugation (permeable). As expected from the available information in cells and our studies of purified Cx26 HC produced in Sf9 cells, the HCs produced in bacteria were permeable to AF350 (molecular weight 349Da), but not to AF647 (molecular weight 1300Da) (Figures 3A and 3B).

**Figure 4:**
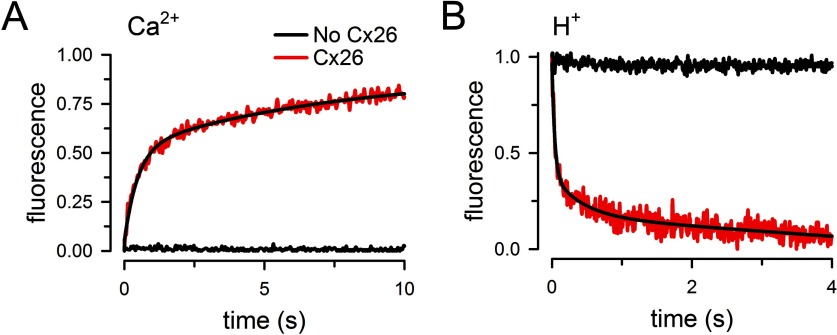
Permeability of purified Cx26 HCs to Ca^2+^ and H^+^ (**A**) Rate of Ca^2+^ influx into liposomes containing purified Cx26 HCs. Proteoliposomes (red trace, Cx26) and liposomes (black trace, no Cx26) were exposed to a 500 μM [Ca^2+^] gradient. Records from Cx26-proteoliposomes or liposomes in the absence of [Ca^2+^] gradient were indistinguishable and did not show a change in fluorescence (similar to the black trace). The black line superimposed to the red record is a multi-exponential fit to the data. The record is representative of 22 measurements in six independent experiments. (**B**) Rate of pH change in Cx26 proteoliposomes. Proteoliposomes (red trace, Cx26) and liposomes (black trace, No Cx26) were exposed to a pH decrease from 7.6 to 6.4. Records from Cx26-proteoliposomes or liposomes in the absence of pH gradient did not show a change in fluorescence (similar to the black trace). The black line superimposed to the red trace is a multi-exponential fit to the data. The record is representative of 22 measurements in four independent experiments.

We also determined permeability to ATP by measuring the fraction of the ATP retained inside liposomes pre-loaded with the nucleotide. For these experiments, extraliposomal ATP was removed by gel filtration in an ATP-free buffer and the nucleotide retained in the liposomes was measured by luminescence as described [22]. Figure 3(C) shows that ATP permeates through the purified Cx26 HCs. We have also shown that Cx26 HCs purified from Sf9 cells are permeable to Ca^2+^ and H^+^ equivalents (H^+^/OH^−^ and protonated/unprotonated HEPES) [22]. For the Ca^2+^ transport assay, we loaded the liposomes with the low-affinity Ca^2+^-sensitive fluorescent probe Fluo-5N and estimated Ca^2+^ influx into the liposomes from the rate of increase in Fluo-5N fluorescence upon increasing free-[Ca^2+^] from <10 nM to 500 μM by rapid mixing in a stop-flow cell. Typical records are shown in Figure 4(A), where an increase in Fluo-5N emission upon rising free-[Ca^2+^] was observed only in the proteoliposomes (red trace). Increasing [Ca^2+^] in liposomes did not elicit net Ca^2+^ influx (black trace). The rate of increase in intraliposomal [Ca^2+^], normalized to a 1 mM Ca^2+^ concentration gradient, was 2.88±0.82 mM/s (*n*=6), as calculated from the increase in Fluo-5N emission and the dependence of Fluo-5N emission on [Ca^2+^], as described [22]. H^+^ transport was evaluated in liposomes containing traces of a phospholipid labelled with fluorescein at the headgroup [22]. As shown in Figure 3(B), lowering pH from 7.6 to 6.4 in a stop-flow cell produced a fast reduction in fluorescein emission only in the liposomes containing Cx26 that were exposed to the pH gradient (red trace). From previous work [22], we know that the decrease in pH produces a rapid quenching of fluorescence from fluorescein on the outer leaflet, similar in liposomes and proteoliposomes (∼50% of the total). This change is very fast and becomes part of the baseline in stop-flow experiments. The slower decrease in intraliposomal pH due to influx of H^+^ through the HCs quenches the emission from fluorescein in the inner leaflet. The latter is followed in the stop-flow experiments and is observed only in the proteoliposomes (red trace). From the rate of decrease in fluorescence and the dependence of fluorescein emission on pH, we calculated a decrease in intraliposomal pH from 7.60 to 7.42±0.01 in 10 ms (*n*=4). From the concentrations of protonated [HEPES] (using a p*K*_a_ of 7.55), the calculated rate of transport of H^+^ equivalents, normalized to a 1 mM HEPES concentration gradient, was 21±1 mM/s (*n*=4). Since the rate of H^+^ transport probably exceeds that of HEPES and the [H^+^] gradient is 373 nM, the above value is only a minimum estimate. The most important observations are that the rates of Ca^2+^ and H^+^ transport are not different from those calculated for Cx26 HCs purified from Sf9 cells [22]. These data also confirm the permeability of Cx26 HCs to Ca^2+^ and ATP and support the idea that Cx26 HCs have a role in Ca^2+^ influx and ATP efflux under physiological and/or pathophysiological conditions [10–13,32,33].

The bacterial expression–purification system in the present study yields purified Cx26 in milligram amounts and equivalent purity to those obtained using the baculovirus–insect-cell expression system. Moreover, the purified Cx26 HCs obtained from *E. coli* are functionally indistinguishable from those formed by Cx26 purified from Sf9 cells [22]; they show the expected permeability properties: permeability to ‘large’ hydrophilic solutes (sucrose, ATP and AF350) and ‘small’ ions (Ca^2+^, H^+^, K^+^, Cl^−^) and impermeability to ‘larger’ hydrophilic solutes (AF647, Fluo-5N) [4,22].

**Figure 5:**
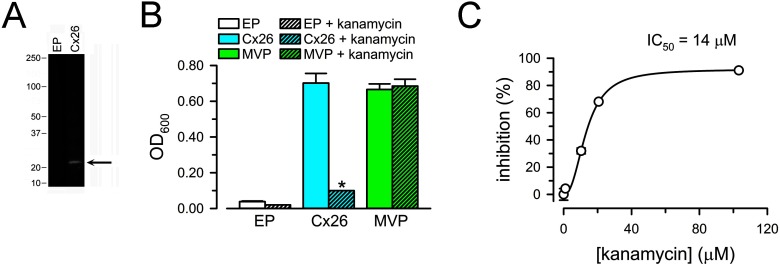
Functional assay of Cx26 HCs in intact bacterial cells (**A**) Expression of Cx26 in LB2003 cells. EP: cells transformed with pQE-60 and grown in high-K^+^ medium (they do not grow in 4 mM [K^+^] medium). Cx26: LB2003 cells transformed with pQE–Cx26. Equivalent amounts of lysed cells were subjected to SDS/PAGE and probed with an antibody against the intracellular loop of Cx26. The two lanes are from the same blot. The arrow points to Cx26 and the labels on the left indicate the position of molecular weight markers (in kDa). (**B**) Growth complementation by expression of Cx26. The experiments were performed under the conditions described in panel (**A**), except that the cells were grown in NLM with 4 mM [K^+^] at 30°C. Complementation by Cx26 was also observed at 37°C, but the signal-to-background ratio was better at 30°C. Growth was determined in the absence (plain bars) or presence of kanamycin (50 μg/ml; 103 μM; hatched bars). EP and Cx26 were defined in (**A**); MVP: LB2003 cells transformed with MVP DNA cloned into pJ404. Data are means ± S.E.M. of 12 measurements in four independent experiments for each condition. *P<0.001 for the decrease in *A*_600_ compared with the corresponding condition in the absence of kanamycin. (**C**) Dependence of the inhibition of Cx26-dependent growth complementation on [kanamycin]. The line corresponds to a fit of the data to the Hill's equation (the Hill coefficient was ∼2). Circles correspond to means ± S.E.M. (*n*=3 for each concentration); S.E.M.s smaller than the symbols are not shown.

The experiments described above indicate that purified human Cx26 HCs expressed in bacteria are functional. Therefore, it may be possible to develop a functional HC assay in the intact cells that will serve as bases for a future high-throughput screening assay for the discovery of HC blockers. Connexin HCs have been proposed as drug targets [34–37], but commonly used HC inhibitors display low affinity and selectivity [38,39]. In addition, there is no evidence that they act by direct binding to the HCs, as opposed to working by indirect mechanisms. For the studies in live bacteria, we used LB2003 cells, which are deficient in K^+^ uptake mechanisms and do not grow in low-[K^+^] medium [24,28]. The cells transformed with human Cx26 DNA cloned into the pQE-60 plasmid expressed Cx26 (Figure 5A) and grew in 4 mM [K^+^] medium, whereas those transformed with the empty plasmid did not (Figure 5B). For these studies, we compared the complementation by Cx26 HCs with that obtained by expression of MVP (Methanococcus jannaschii voltage-gated potassium channel). MVP is a hyperpolarization-activated K^+^ channel that displays high open probability at the large cell-negative membrane voltages characteristic of *E. coli* [40].

Very recently, aminoglycosides have been shown to inhibit connexin HCs with better affinity than most inhibitors and without effect on GJCs [41]. The data in Figure 5(C) show that kanamycin inhibits the growth complementation in cells expressing Cx26 HCs, with an IC_50_ of 14 μM. The effect was not related to the antibiotic effect of kanamycin because LB2003 cells are resistant to kanamycin [24] and there was no effect on growth complementation by expression of MVP (Figure 5C). Ototoxicity is among the most common adverse effects of aminoglycosides, but its mechanism is still unclear [42]. It is presently unknown whether the inhibition of Cx26 HCs is associated with the ototoxicity.

In additional studies, we found that Cx26 purified from LB2003 cells was functional; the fraction of the ATP retained inside Cx26 liposomes pre-loaded with the nucleotide was 10±1% of the value in liposomes without Cx26 (*n*=3), a value indistinguishable from that measured in liposomes containing Cx26 purified from XL10-Gold cells (Figure 3C).

In summary, we presented a robust *E. coli*-based Cx26 expression system that yields purified and functional Cx26 HCs in amounts equivalent to those obtained in insect cells. One limitation of the bacterial expression system is in the post-translational modifications. However, connexins are not glycosylated and there is no clear evidence of direct regulation of Cx26 by post-translational modifications. This is in marked contrast with other isoforms that contain a regulatory C-terminal domain, such as Cx43 [43]. The observation that the Cx26 HCs purified from bacteria have similar properties than those expressed in eukaryotic cells indicates that the bacterial expression system will be useful to study the basic properties of wild-type and mutant HCs and will be a useful complement to others currently in use. There are few, if any, examples of expression in *E. coli* of properly folded and functional human recombinant membrane proteins with milligram per litre culture yields. This new expression–purification system has the potential to increase the pace of structural and functional studies of connexins in a major way.
